# Predictive value of obsessive-compulsive drinking scale (OCDS) for outcome in alcohol-dependent inpatients: results of a 24-month follow-up study

**DOI:** 10.1186/1747-597X-6-14

**Published:** 2011-06-28

**Authors:** Peggy Schmidt, Claudia Helten, Michael Soyka

**Affiliations:** 1 Psychiatric Hospital, Ludwig-Maximilians-University, Nußbaumstr. 7, 80336 Munich, Germany; 2 Private Hospital Meiringen, Willingen, 3860 Meiringen, Switzerland

**Keywords:** Treatment, alcohol, alcoholism, craving, OCDS, outcome

## Abstract

**Background:**

The present study examined whether craving as measured by the obsessive-compulsive drinking scale (OCDS) predict long-term outcome in alcohol-dependent inpatients.

**Methods:**

This was a 24-month prospective, observational study in 198 alcohol-dependent inpatients treated under standardized conditions. The primary outcome criterion was abstinence, defined as no subjective report or objective indication of alcohol consumption since discharge from treatment. The patients self-rated their craving for alcohol at the 6- and 12-month follow-ups by using the German version of the OCDS, which measures obsessive and compulsive aspects of craving. Univariate and logistic regression analyses with covariates were performed.

**Results:**

Of the 104 patients interviewed at the 24-month follow-up, 60% (*n *= 62) were abstinent. We found significant associations between total OCDS scores at 6 months and outcome at 12 months and between total OCDS scores at 12 months and outcome at 24 months: the higher the OCDS total score at one follow-up evaluation, the less likely patients were to be abstinent at the subsequent one. The same association was found for each of the two OCDS subscales, control and consequences and drinking obsessions.

**Conclusions:**

These results support earlier findings that OCDS scores can predict outcome in alcohol-dependent patients. This information can be used for the timely development of protective resources. Hence, decisions over the use of resources can be made on the basis of objectified parameters to develop a personalized treatment concept. Consequently, economic considerations can induce a reduction of high medical costs.

## Background

Craving is a multidimensional construct that has both positive and negative reinforcement properties and plays a key role in relapse to alcohol consumption. It comprises thoughts about alcohol and urges to drink alcohol and is associated with negative affect, depressed mood, distress or withdrawal symptoms (for review see Abrams) [1-7]. There is plethora of research on different forms of craving in substance use disorders [8-12], with some studies indicating that subjective craving is predictive of treatment outcome [13-19]. Patients in remission are particularly prone to alcohol-related cues or stress that may induce craving [20-23].

The obsessive-compulsive drinking scale (OCDS) [24] is the most widely used multi-factorial self-rated craving scale in alcohol research and treatment. The OCDS measures various aspects of craving for alcohol, including the compulsive urge to drink alcohol, continuous thoughts about alcohol and the struggle to control the urgency. The scale is a modified version of the Yale-Brown Obsessive Compulsive Scale [25,26] and aims to measure both obsessive and compulsive aspects of craving. The 14 items of the scale are divided into two subscales, control and consequences (CC) and drinking obsessions (DO). The OCDS has been shown to be a valid self-rated instrument with good test-retest reliability and internal consistence [24,27]. There are several validated translations with good reliability and construct validity, including a German version [6,28-31]. The studies that examined the construct, concurrent and discriminate validity of the OCDS [24,27,32-35] were reviewed by Connor et al. [36], who also performed a further validation study and reported that neither the factor scores nor the total OCDS score was related to baseline alcohol problems or consumption.

The predictive value of OCDS scores for treatment outcome has been demonstrated in some but not all previous studies [37,38] and needs further confirmation [35]. Our goal was to examine the association between OCDS scores and outcome in a sample of alcohol-dependent inpatients treated under standardized conditions.

## Methods

### Subjects

The subjects were 198 alcohol-dependent inpatients. From January to December 2003, all patients admitted to the inpatient clinic AHG Clinic Wilhelmsheim, Germany for treatment of alcohol dependence were consecutively recruited into the study at the start of their treatment. The primary study inclusion criterion was an ICD-10 and DSM-IV diagnosis of alcohol dependence. Exclusion criteria were dependence on benzodiazepines or illicit drugs or both, severe physical illness and severe psychiatric disorders such as psychosis or acute suicidality. All patients who entered treatment participated in the study and all gave written informed consent to participate. The study protocol was approved by the local ethics committee, and the study was performed according to the principles laid down in the Declaration of Helsinki.

Patients received a standard alcohol treatment, which lasted for either 8 weeks (for less severe cases) or 12 to 16 weeks (for more severe cases). The treatment includes both psychoanalytical and behavioural approaches and methods. The treatment concept at the clinic follows an abstinence-oriented approach.

### Assessments

This was a prospective, 24-month follow-up study that measured outcome, defined as abstinence.

Assessments of diagnostic criteria for disorders according to DSM-IV and ICD-10 were made by the Munich Composite International Diagnostic Interview [39,40]. Further variables relevant for the analyses were recorded in structured, face-to-face interviews at the start of the programme (Baseline, T0), at discharge from the treatment unit (T1), and at the 6-month (T2), 12-month (T3) and 24-month (T4) follow-ups. The baseline assessment included demographic variables, past and current psychiatric, medical and substance use-related problems, and drinking parameters. Patients were asked about prior detoxifications, prior alcohol rehabilitation and prior treatments for psychiatric problems, except for alcohol dependence. At discharge, the length of time spent in the programme, mode of discharge from the programme (e.g. successfully completed the programme, left prematurely by choice), and relapses during treatment were recorded. Alcohol consumption was reported using the Timeline Followback interview. Patients completed the German version of the OCDS [31] at T2 and T3.

The interviewers were trained psychologists, physicians and medical students and were not involved in the treatment of interviewed subjects; the project coordinator was not a member of the clinical staff. But, the interviewers as well as the project coordinator were in contact with the therapists. For further details see Soyka and Schmidt [41].

Table 1 summarizes the variables, assessment instruments and assessment times.

**Table 1:** Variables, assessment instruments and assessment times

T0	T1	T2	T3	T4
Treatment start	At discharge	6 months after discharge	12 months after discharge	24 months after discharge

EuropASI/ patient files: Demographics Psychiatric, medical and substance use problems Drinking parameters	EuropASI/ patient files: Time in treatment Mode of discharge Relapses	Total abstinence during the 6 months Relapse	Total abstinence during the 12 months Relapse	Total abstinence during the 24 months Relapse
		OCDS	OCDS	

### Definition of outcome criterion

The primary outcome criterion was abstinence 6, 12 and 24 months after discharge from treatment. Abstinence was defined according to the definition from Feuerlein and Kuefner [42] as no subjective report or objective indication of alcohol consumption since discharge. This criterion was used as the dependent variable in the data analyses.

For data analysis, patients were divided into two groups: those who were personally interviewed at the 24-month follow-up and those who did not attend the 24-month follow-up interview.

### Data analysis

Statistical analyses were performed using SPSS for Windows [43].

Absolute and relative frequencies, means and standard deviations (SD) were calculated for data description. Univariate comparisons of responders and non-responders were performed by using the likelihood ratio statistic (for alternative and categorial data), the Mann-Whitney U test (for ordinal data), and the Kolmogorov-Smirnov test (for metric data). The predictive value of the OCDS scores was analyzed with logistic regression analyses. The variables which differed between responders and non-responders were inserted as covariates.

All statistical tests were two tailed. A *p *value of less than 0.05 was considered to be statistically significant. We performed one analysis that only included the data of patients who were personally interviewed at the 24-month follow-up and another that also included the data from those who did not attend the 24-month interview; we repeated these two analyses after applying the OCDS modification for longitudinal studies to the data (see Nakovics et al. for further details [44]), making a total of four sets of analyses. There were no significant differences between the results of these four sets of analyses.

## Results

### Subject characteristics and outcome

The subject characteristics are summarized in table 2. One hundred and ninety-eight patients were enrolled in the study and 104 patients attended the 24-month follow-up. At admission, the mean age of the patients was 45.6 (*SD *= 7.4) years. The average duration of alcohol dependence was 11.4 (*SD *= 8.1) years, and the mean age of onset of alcohol dependence 34.0 years (*SD *= 9.1).

**Table 2:** Baseline and T1 characteristics of subjects - shown for the total sample and according to drinking status (abstinent or non-abstinent) at the 24-month follow-up (T4)

	Total sample	Patients who responded at T4		Difference abstinent vs. non-abstinent
	(*n *= 104)	abstinent at T4 (*n *= 62)	non-abstinent at T4 (*n *= 42)	

*Baseline*				
Age (*M, SD*)	45.6 (7.4)	46.2 (7.4)	44.7 (7.4)	Z = 0.6; p = 0.93^a^
Sex (*n, %*)				LR(1, n = 104) = 0.8; p = 0.38^b^
Male	77 (74)	44 (71)	33 (79)	
Female	27 (26)	18 (29)	9 (21)	
Without secondary school qualifications (*n, %*)	3 (3)	2 (3)	1 (2)	LR(1, n = 104) = 2.3; p = 0.80^b^
Without professional training (*n, %*)	29 (28)	17 (27)	12 (29)	LR(1, n = 104) = 4.3; p = 0.37^b^
Employment status (*n, %*)				LR(4, n = 104) = 9.7; p = 0.05*^b^
Employed	65 (63)	42 (68)	23 (55)	
Unemployed	35 (34)	16 (26)	19 (45)	
Retired	4 (4)	4 (7)	0	
Residential situation (*n, %*): Living ...				LR(5, n = 104) = 10.5; p = 0.61^b^
alone	35 (34)	15 (24)	20 (48)	
with parents	4 (4)	1 (2)	3 (7)	
with children	5 (5)	4 (7)	1 (2)	
with cohabitant and with/without children	59 (57)	41 (66)	18 (43)	
with friends	1 (1)	1 (2)	0	
Marital status (*n, %*)				LR(5, n = 104) = 8.4; p = 0.14^b^
Single	21 (20)	10 (16)	11 (26)	
Married	47 (45)	32 (52)	15 (36)	
Separated	5 (5)	2 (3)	3 (7)	
Divorced	27 (26)	14 (23)	13 (31)	
Widowed	4 (4)	4 (7)	0	
Age of onset of alcohol use (years: *M, SD*)	15.1 (3.9)	15.3 (4.3)	14.8 (3.3)	Z = 0.5; p = 0.97^a^
Age of onset of regular alcohol use (years: *M, SD*)	22.4 (7.3)	22.8 (7.4)	21.9 (7.2)	Z = 0.7; p = 0.78^a^
Age of onset of alcohol dependence (years: *M, SD*)	34.0 (9.1)	34.7 (9.3)	32.9 (8.8)	Z = 0.8; p = 0.54^a^
Duration of alcohol dependence (years: *M, SD*)	11.4 (8.1)	11.5 (8.8)	11.2 (7.0)	Z = 1.1; p = 0.63^a^
Daily alcohol intake (g/day: *M, SD*)	176.8 (140.7)	156.0 (101.9)	207.5 (180.8)	Z = 0.8; p = 0.19^a^
Number of previous treatments (*M, SD*) for				
alcohol				
detoxification	3.7 (8.6)	2.9 (8.4)	4.7 (9.0)	U = 996.5; p = 0.03*^c^
rehabilitation	0.2 (0.5)	0.1 (0.2)	0.4 (0.7)	U = 1006.0; p = 0.002**^c^
psychiatric problems	1.3 (6.1)	0.9 (4.6)	1.9 (8.0)	U = 1266.5; p = 0.75^c^
medical problems	3.3 (3.0)	2.8 (1.9)	4.0 (4.1)	U = 1160.0; p = 0.34^c^

*T1*				
Repeated alcohol relapse during treatment (*n, %*)	4 (4)	0	4 (10)	LR(1, n = 104) = 7.5; p = 0.01**^b^
Treatment drop out (*n, %*)	9 (9)	2 (3)	7 (17)	LR(1, n = 104) = 5.7; p = 0.03*^b^

^a ^Kolmogorov-Smirnov test, ^b ^Likelihood ratio statistic, ^c ^Mann-Whitney U test.

*p < 0.05; **p < 0.01.

Of the patients interviewed at the 24-month follow-up (T4; *n *= 104), 72% (*n *= 75) had been continuously abstinent until 6 months after treatment discharge (T2), 67% (*n *= 70) until the 12-month follow-up (T3), and 60% (*n *= 62) until T4. There are no significant differences in the baseline and T1 characteristics between the 94 patients who did not attend the 24-month follow up and the 104 patients who attended this follow up.

Significant differences were found at T4 between abstinent (*n *= 62) and non-abstinent patients (*n *= 42) for employment status: 42 (97.7%) of the patients abstinent at T4 were employed at T0 but only 23 (55%) of the non-abstinent patients. Furthermore, the non-abstainers had participated in more previous alcohol detoxifications and more previous alcohol rehabilitations than the abstainers. The non-abstainers had repeated alcohol relapses during the treatment period, and more patients of this group dropped out.

### Association between the OCDS scores and outcome

**Figure 1:**
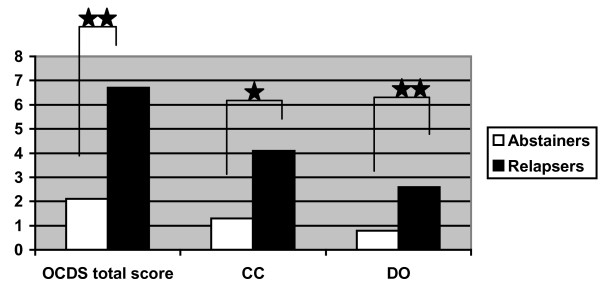
**12-month follow-up**. Differences in total OCDS score, control and consequences subscore (CC) and drinking obsession subscore (DO) at the 6-month follow-up between patients who were abstainers (*n *= 62) and those who were non-abstainers (*n *= 42) at the 12-month follow-up. Logistic regression analyses: 1. column Wald = 7.0; df = 1; p = 0.01 2. column Wald = 6.6; df = 1; p = 0.05 3. column Wald = 6.5; df = 1; p = 0.01. *p < 0.05; **p < 0.01.

**Figure 2:**
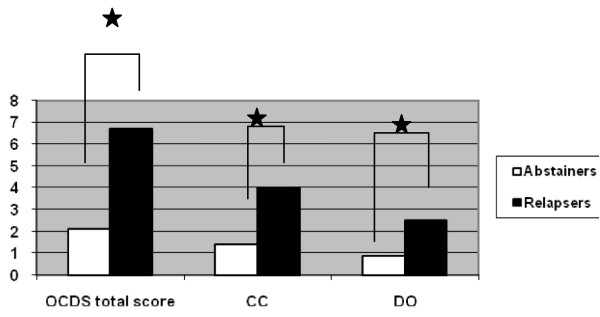
**24-month follow-up**. Differences in total OCDS score, control and consequences subscore (CC) and drinking obsession subscore (DO) at the 12-month follow-up between patients who were abstainers (*n *= 62) and those who were non-abstainers (*n *= 42) at the 24-month follow-up. Logistic regression analyses: 1. column Wald = 6.3; df = 1; p = 0.05 2. column Wald = 5.4; df = 1; p = 0.05 3. column Wald = 5.1; df = 1; p = 0.05. *p < 0.05.

As to be seen in figures 1 and 2, associations were found between the OCDS scores at T2 and outcome at T3 as well as between the scores at T3 and outcome at T4 for both OCDS subscales and the total OCDS score. The mean 6-month OCDS scores of patients abstinent or non-abstinent at T3 were as follows: 1.3 in abstainers vs. 4.1 in non-abstainers (*OR *= 0.8, *p *< .05, 95% CI = 0.7, 0.9) in the CC subscore; 0.8 in abstainers vs. 2.6 in non-abstainers (*OR *= 0.8, *p *< .01, 95% CI = 0.6, 0.9) in the DO subscore; and 2.1 in abstainers and 6.7 in non-abstainers (*OR *= 0.8, *p *< .01, 95% CI = 0.7, 0.9) in the total score. The mean 12-month OCDS scores of patients abstinent or non-abstinent at T4 were as follows: 1.4 in abstainers vs. 4 in non-abstainers (*OR *= 0.8, *p *< .05, 95% CI = 0.7, 0.9) in the CC subscore; 0.9 (abstainers) vs. 2.5 (non-abstainers) in the DO subscore (*OR *= 0.8, *p *< .05, 95% CI = 0.6, 0.9); and 2.1 (abstainers) vs. 6.7 (non-abstainers) in the total score (*OR *= 0.8, *p *< .05, 95% CI = 0.7, 0.9).

## Discussion

Our findings indicate that in alcohol-dependent inpatients being treated under standardized conditions in a specialized alcohol inpatient facility, OCDS scores 6 months after discharge are predictive for the 12-month outcome and OCDS scores 12 months after discharge are predictive for the 24-month outcome. Concerning significant results in both subscales, it seems that obsessions as well as control/consequences about alcohol are connected closely with alcohol relapse.

Our findings are in line with other studies reporting that the magnitude of craving is predictive for drinking outcome in alcohol-dependent patients [17,27,35,37,45,46].

Richardson et al [17] randomized 169 patients (70 male, mean age 45) who were treated across three outpatient clinics in Sydney, Australia to receive acamprosate, naltrexone or placebo. They found craving to be a significant predictor of daily drinking during treatment in independence of baseline depression and dependence severity.

Anton et al [27] assessed 41 alcohol-dependent individuals weekly with the OCDS during a 12-week pharmacologic and cognitive-behavioural treatment. The OCDS total and the subscale scores were significantly higher in subjects who had relapsed during the time after the assessment.

Roberts et al [35] studied 132 alcohol dependent patients seeking outpatient treatment. Patients received either 50 mg naltrexone or placebo daily for 12 weeks and attended 12 sessions of cognitive behavioural therapy. The authors suggested the OCDS may better predict shorter term drinking outcomes than prolonged outcomes as each of the OCDS subscale scores predicted the hazard for heavy drinking during the following treatment week.

Bottlender and Soyka [37] reported on 103 patients attended an intensive outpatient treatment program for around 12 month. Patients who relapsed during the treatment phase had significantly higher total OCDS scores as well as higher scores on the subscales 'obsessions' and 'drinking control and consequences' compared to abstinent patients. Furthermore, major relapse was predicted by the total OCDS score and the subscale 'obsessions'.

Gordon et al [45] reported on 218 alcohol-dependent patients admitted to two separate residential addiction treatment programs. They found that days craving reported in the week prior to discharge predicted alcohol use at the three-month follow-up.

Kranzler et al [46] initiated a study with 127 alcohol depended subjects who attended a 12-week outpatient pharmacotherapy trial with a 3-month follow-up period. The predictive validity of the OCDS was not found to be significant but was a tendency.

There is some debate as to whether the OCDS includes questions that may not represent the core concept of craving and therefore requires changes [29,44,47]. Still, taken together, our findings suggest that craving as defined and measured by the OCDS items is indeed relevant for predicting long-term outcome in patients. Data from this study further emphasize the role of craving for treatment and outcome in alcohol dependence.

Allocating patients to different treatment settings according to their symptom profile and prediction of response is a major but difficult clinical task and results of studies are conflicting [48]. Craving has been identified as one of the key symptoms in alcohol dependence and as a major cause of relapse to alcohol [4,14,34,49], craving is similarly relevant in other forms of substance use, especially cocaine [13,18]. Craving can be but does not have to be cue related [12,16,50] and can be linked to different positive and negative affective stimuli, cognitive processes and especially stress [3,7,9,11,51]. The interrelationship between craving and relapse is unclear and many relapses occur without any clear subjective experience of craving. Still, there is robust evidence for a predictive role of craving for relapse to heavy drinking and many treatment studies use craving scales at least as secondary outcome parameters [14,16,17,19].

The OCDS aims to measure key features of craving [24] and is by far the most frequently used scale in this respect. It was developed on the basis of two theoretical obsessive and compulsive dimensions of alcohol craving [25,26] and is divided into two subscales, obsessions (drinking obsessions; DO) and compulsions (control and consequences; CC). Its predictive value is still a matter of debate [35,36,38]. Previously, we demonstrated that the OCDS total score and each of the two subscores are predictive for 12-month follow-up after outpatient treatment for alcohol dependence [37]. Kranzler et al. [46] also demonstrated that a higher OCDS score is predictive for a worse outcome.

There are several limitations to this study. First, no biological markers (such as CDT or GGT) were used to verify outcome and no collateral informants were available. Still, patients were repeatedly seen over a two-year period and personally interviewed, so that the results can be assumed to be reliable. Second, no other craving scales were used to cross-verify results. However, many studies indicate that the OCDS has well to excellent values for validity, as discussed above. As it is another situation if the patients are in treatment compared to the follow up time, we just used the OCDS at time point T2 and T3. Finally, only 104 out of 198 patients who entered inpatient treatment could be followed up by personal interview after 2 years. Nevertheless, this rate is acceptable for follow-up studies in alcoholic patients.

## Conclusions

The results of this study further support that OCDS scores may have predictive value in alcohol-dependent patients and that the OCDS may also be a useful tool in clinical practice to identify patients at risk for relapse. To avoid high follow up costs for further treatments, patients with higher relapse potential are detectable in an earlier stage. The information is useful for the timely development of protective resources. Decisions over the use of resources can be made on the basis of objectified parameters to develop a personalized treatment concept. In this manner, economic considerations can induce a reduction of high medical costs.

## Competing interests

The authors declare that they have no competing interests.

## Authors' contributions

PS performed the statistical analysis and drafted the manuscript. CH helped to draft the manuscript. MS conceived the study, participated in its design and coordination and helped to draft the manuscript. All authors read and approved the final manuscript.
